# Spherical Al-MCM-41 Doped with Copper by Modified TIE Method as Effective Catalyst for Low-Temperature NH_3_-SCR

**DOI:** 10.3390/molecules26061807

**Published:** 2021-03-23

**Authors:** Aleksandra Jankowska, Andrzej Kowalczyk, Małgorzata Rutkowska, Marek Michalik, Lucjan Chmielarz

**Affiliations:** 1 Faculty of Chemistry, Jagiellonian University in Kraków, Gronostajowa 2, 30-387 Krakow, Poland; a.jankowska@doctoral.uj.edu.pl (A.J.); kowalczy@chemia.uj.edu.pl (A.K.); rutkowsm@chemia.uj.edu.pl (M.R.); 2 Institute of Geological Sciences, Jagiellonian University in Kraków, Gronostajowa 3a, 30-387 Krakow, Poland; marek.michalik@uj.edu.pl

**Keywords:** silica-alumina spherical MCM-41, copper, template ion-exchange method, NH_3_-SCR

## Abstract

Aluminum containing silica spherical MCM-41 was synthesized and modified with copper by the template ion-exchange method (TIE) and its modified version, including treatment of the samples with ammonia solution directly after template ion-exchange (TIE-NH_3_). The obtained samples were characterized with respect to their chemical composition (ICP-OES), structure (XRD), texture (low temperature N_2_ sorption), morphology (SEM-EDS), form and aggregation of deposited copper species (UV-vis DRS), reducibility of copper species (H_2_-TPR), and surface acidity (NH_3_-TPD). The deposition of copper by the TIE-NH_3_ method resulted in much better dispersion of this metal on the MCM-41 surface comparing to copper introduced by TIE method. It was shown that such highly dispersed copper species, mainly monomeric Cu^2+^ cations, deposited on aluminum containing silica spheres of MCM-41, are significantly more catalytically effective in the NH_3_-SCR process than analogous catalysts containing aggregated copper oxide species. The catalysts obtained by the TIE-NH_3_ method effectively operated in much broader temperature and were less active in the side process of direct ammonia oxidation by oxygen.

## 1. Introduction

Nitrogen oxides, NO and NO_2_, belong to the major pollutants present in exhaust gases emitted by thermal power plants, industrial furnaces, and motor vehicles, and are the result of fuel combustion. Nitric oxide (NO), which is the primary product can be formed by three possible mechanisms [1]: (1) thermal mechanism: reaction of nitrogen and oxygen in gas phase of high temperature zone of boiler; (2) fuel mechanism: reaction of nitrogen bound in the fuel with oxygen from gas phase of high temperature zone of boiler; (3) prompt mechanism: rapid reaction of atmospheric nitrogen with hydrocarbon radicals. Nitrogen dioxide (NO_2_) is formed by NO oxidation outside of the high-temperature zone of boiler [2]. Nitrogen oxides contribute to the formation of acid rain and cause a wide range of environmental concerns. NO_x_ is the main constituent in the formation of ground-level ozone, which causes respiratory problems. Such respiratory problems may result from exposure to NO_2_ by itself but can be caused by NO_x_ reacting to form airborne nitrate particles or acid aerosols resulting in the similar effects. Another side effect of NO_x_ emission is deterioration of water quality by overloading the water with nutrients causing an overabundance of algae [3]. The most effective technology for the conversion of NO_x_ in exhaust gases emitted by stationary sources is its selective catalytic reduction with ammonia resulting in gaseous nitrogen (NH_3_-SCR). This technology effectively operates in the temperature range of 300–400 °C in the presence of the catalysts based on V_2_O_5_-TiO_2_ metal oxide systems [4]. The main advantages of these catalysts are their high selectivity to nitrogen and tolerance for SO_x_ [5]. While the main disadvantages are narrow temperature range of effective operation and relatively high volatility of vanadium [6]. Therefore, the studies focused on development of new catalysts for the NH_3_-SCR process are still conducted. One of the main goals is development of the catalysts operating at low temperatures (below 250 °C), what could result in retrofitting of the installations for gas purification in thermal power plants and industrial furnaces. Nowadays, NH_3_-SCR units in such installations are located upstream of electrostatic precipitator (ESP), and therefore monolithic NH_3_-SCR converters operate with a dusty gas stream (high-dust SCR). In such case there is a risk of monolith channels plugging by particles of dust present in flue gases. The changing of the ESP and NH_3_-SCR order in the gas exhaust installation should result in protection of the monolithic NH_3_-SCR unit from plugging by dust particles. The problem is related to relatively low temperatures of ESP unit operation and therefore for such reconfiguration of the ESP and NH_3_-SCR modules, development of the effective catalysts operating at temperature of 250 °C or even lower is necessary [7].

Zeolites modified with copper belong to the group the most promising catalysts for the low-temperature NH_3_-SCR process [8,9,10]. It is not surprising considering their relatively high specific surface area, ion-exchange properties enabling deposition of copper in the highly dispersed form as well as surface acidity resulting from the presence of aluminum in the zeolite framework. The recent studies of the authors have shown the very promising activity of silica MCM-41 modified with copper by template ion-exchange (TIE) method in the low temperature NH_3_-SCR process [11]. Moreover, it was shown that the dispersion of deposited copper species can be improved by post-treatment of the copper modified silica samples, directly after TIE, with ammonia solution, which significantly improved their low-temperature activity in the NH_3_-SCR process [11]. Further improvement of the catalytic performance was obtained by the replacement of classical MCM-41 for its spherical form [12], possibly due to improved internal diffusion of reactants in shorter channels. Thus, similarly to zeolites, mesoporous silica is characterized by high specific surface area and porosity. Moreover, the developed modified TIE method results in the deposition of copper in highly dispersed forms, also in the case of high loadings of this metal. On the other side, in contrast to zeolites, up-till-now only the pure silica supports were used for catalysts preparation by modified TIE method [11,12]. Thus, the next step of the studies, presented in this manuscript, has been focused on the analysis of aluminum containing silica spherical MCM-41 as support of the catalysts for the NH_3_-SCR process.

## 2. Results and Discussion

**Figure 1:**
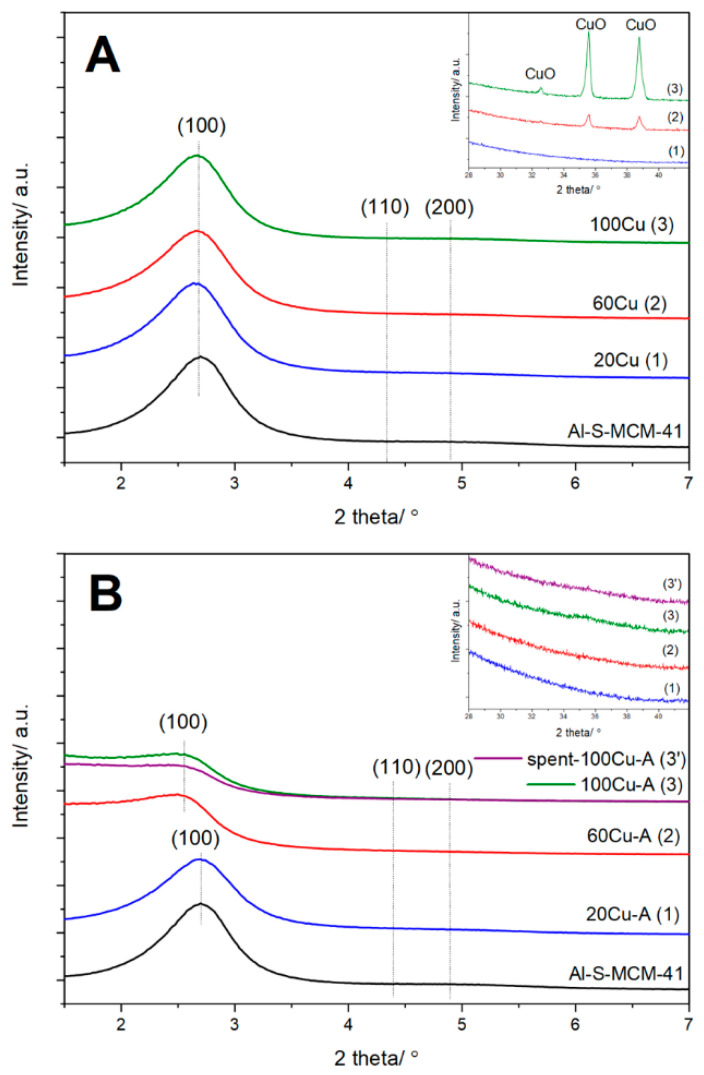
Powder X-ray diffractograms of the Al-S-MCM-41 sample and its modifications with copper obtained by TIE (**A**) and TIE-NH_3_ (**B**) methods.

The X-ray diffractograms of spherical MCM-41 and its modifications with copper are presented in Figure 1. The three diffraction peaks, (100), (110) and (200), characteristic of MCM-41 should be expected in diffractograms. However, only the (100) reflection is present in diffractograms of the samples. In positions characteristic of the (110) and (200) reflections only slightly increased baseline level can be observed. Thus, the incorporation of aluminum into silica walls of spherical MCM-41 decreased ordering of pores in comparison to pure silica spherical MCM-41 [12]. Modification of alumina-silica spherical MCM-41 (Al-S-MCM-41) with copper by template ion-exchange (TIE) did not result in any significant changes of diffractograms (Figure 1A), indicating stability of the MCM-41 porous structure under conditions of TIE procedure. On the other side, diffraction peaks characteristic of CuO crystallites appeared in diffractograms of the samples modified with copper (Figure 1A, insert). The intensity of these reflections is higher for the samples with larger copper loading. In the case of the samples modified with copper by TIE method followed by their treatment with ammonia the intensity of the (100) reflection decreased, especially for the samples with higher copper loadings—60Cu-A and 100Cu-A (Figure 1B). This effect could be explained by partial destruction of the ordered porous structure in basic conditions of the post TIE treatment of the samples with ammonia (TIE-NH_3_).

The concentrations of ammonia solutions used for such treatment increased with an increase in copper loading (Table 1), thus the most destructive effect of the basic ammonia solution was observed for the 60Cu-A and 100Cu-A samples. In contrast to the samples modified by TIE method, in diffractograms of the samples doped with copper by the TIE-NH_3_ method, any diffraction peaks characteristic of CuO crystallites were identified (Figure 1B, insert). Thus, the treatment of the copper modified samples with ammonia solution prevents the formation of aggregated copper oxide species.

**Figure 2:**
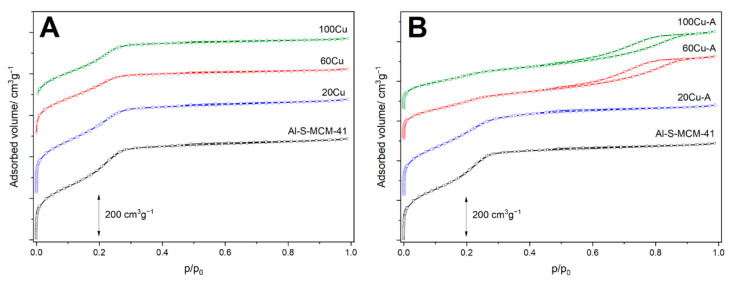
N_2_ adsorption-desorption isotherms of the Al-S-MCM-41 sample and its modifications with copper obtained by TIE (**A**) and TIE-NH_3_ (**B**) methods.

Textural properties of the samples were analysed by low-temperature nitrogen sorption. The N_2_-sorption isotherms of Al-S-MCM-41 and its modifications with copper, presented in Figure 2, are classified as type IV according to the IUPAC standards and are characteristic of mesoporous materials, such as MCM-41 [13,14]. The characteristic feature of the isotherm recorded for Al-S-MCM-41 is a steep increase in nitrogen uptake at a relative pressure of 0.15–0.30 assigned to the capillary condensation of N_2_ molecules inside mesopores. Deposition of copper into Al-S-MCM-41 by TIE method resulted in a small decrease in this nitrogen uptake indicating decrease of mesopore volume, possibly related to deposition of copper species inside of mesopores or partial plugging of these pores by bulky CuO aggregates (Figure 2A). This effect was significantly more distinct for the samples modified with copper by TIE-NH_3_ method, especially for the samples with larger loadings of this metal, 60Cu-A and 100Cu-A (Figure 2B). As it was shown by XRD analysis (Figure 1A) copper oxide crystallites were not identified in these samples, therefore decrease of mesopore volume is possibly associated with deposition of dispersed copper species inside mesopores as well as partial destruction of the porous structures of the samples in basic conditions of their treatment with ammonia solutions. An increase in nitrogen sorption volume, observed for 60Cu-A and 100Cu-A in the relative pressure range of 0.5–0.9, is possibly related to the presence of larger pores located between sticked silica-alumina spheres. The hysteresis loops observed in adsorption-desorption isotherms belong to H3 category according to the IUPAC classification and are characteristic of non-rigid aggregates of plate-like particles and also for the pore network consisting of large pores, which are not completely filled with pore condensate [13,14].

**Figure 3:**
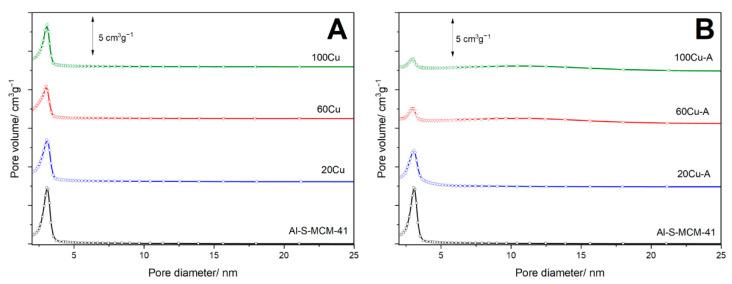
Pore size distributions of the of the Al-S-MCM-41 sample and its modifications with copper obtained by TIE (**A**) and TIE-NH_3_ (**B**) methods.

Pore size distribution (PSD) profiles of the samples, presented in Figure 3, indicate uniform porous structure with the maximum of pore diameter at about 3.0–3.1 nm. Deposition of copper resulted in a decrease of this maximum intensity, indicating deposition of coppers species inside pores. This effect is more distinct for the samples modified by TIE-NH_3_ method, especially for 60Cu-A and 100Cu-A (Figure 3B). In this case, the intensity of the maximum in the PSD profiles is also associated with the partial destruction of the ordered porous structure under conditions of ammonia treatment of the samples. Moreover, in the PSD profiles of 60Cu-A and 100Cu-A broad maxima in the range of 5–22 nm, possibly related to the spaces between condensated spheres, can be found (Figure 3B). Probably, such condensation occurred during the treatment of the samples with ammonia directly after TIE procedure. Probably in a basic solution of ammonia, part of the surface silica was dissolved and then played the role of binder for sphere condensation.

Textural parameters of the samples are compared in Table 2. The specific surface area (SSA) determined for the Al-S-MCM-41 samples is nearly 1200 m^2^ g^−1^, but the deposition of copper resulted in a decrease of SSA. In the series of the samples modified by the TIE method, these changes are significantly lower comparing to the catalysts obtained by the TIE-NH_3_ method, which is possibly related not only to the deposition of copper species inside pores, but also partial destruction of their ordered porous structure under conditions of ammonia treatment. Moreover, the pore volume of the samples decreased after copper deposition, although in this case the effect was more significant for the catalysts obtained by the TIE method. This could be explained by the formation of additional pores by the condensation of spheres under conditions of ammonia treatment for the samples of TIE-NH_3_ series.

The intended molar Si/Al ratio in Al-S-MCM-41 is 20, while real value of this ratio, determined by chemical analysis is 27 (Table 2). Thus, silicon was preferentially incorporated into MCM-41 walls than aluminum. The deposition of copper by TIE methods resulted in a gradual increase in the Si/Al ratio, which for 100Cu reached 37. This effect is possibly attributed to extraction of some aluminum from Al-S-MCM-41 under acidic conditions of CuCl_2_ solution used for TIE procedure. There is not such correlation for the series of the samples obtained by TIE-NH_3_ method (Table 2). In this case, the Si/Al ratios are very similar (25–27) for the samples of this series. A decrease in the silicon content could be explained by their extraction from the samples under conditions of ammonia treatment directly after TIE procedure. It should be noted that the Si/Al molar ratio in Al-S-MCM-41 is the same as in 20Cu and 20Cu-A (Table 2). In the case the concentrations of CuCl_2_ and ammonia solutions used of these samples’ modifications were few times lower than the concentrations of the solutions used for modifications of the other samples. Thus, such diluted solutions were less effective in extraction of aluminum and silicon from the Al-S-MCM-41 support.

**Figure 4:**
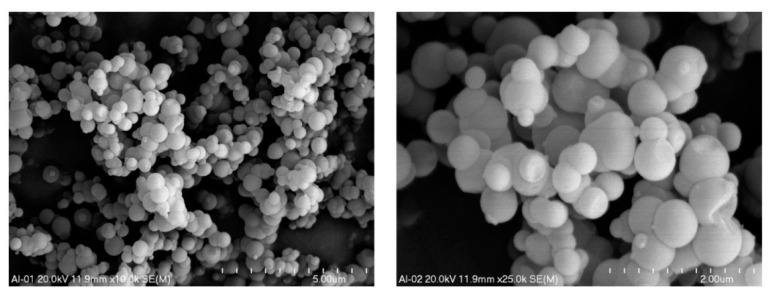
SEM images of spherical silica-alumina MCM-41 at various magnifications.

The morphology of the samples was analysed by scanning electron microscopy (SEM). As it was shown in micrographs recorded for Al-S-MCM-41, this sample is composed of spheres with a diameter of about 200–700 nm (Figure 4). The point chemical analysis made by the EDS method proved the presence of aluminum incorporated into the silica spheres.

**Figure 5:**
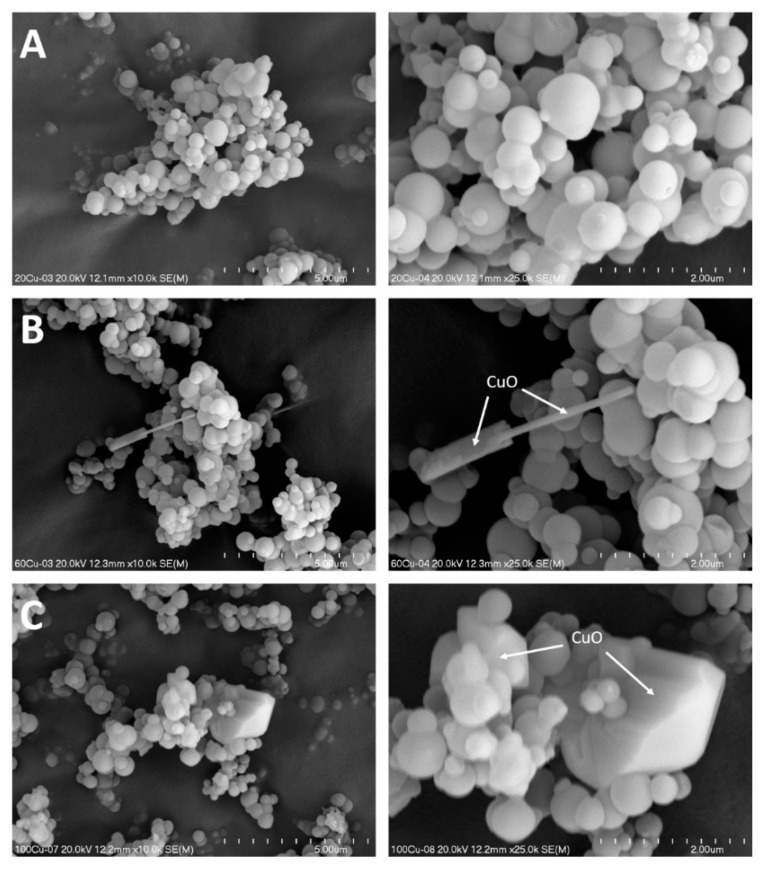
SEM images of the 20Cu (**A**), 60Cu (**B**) and 100Cu (**C**) samples at various magnifications.

**Figure 6:**
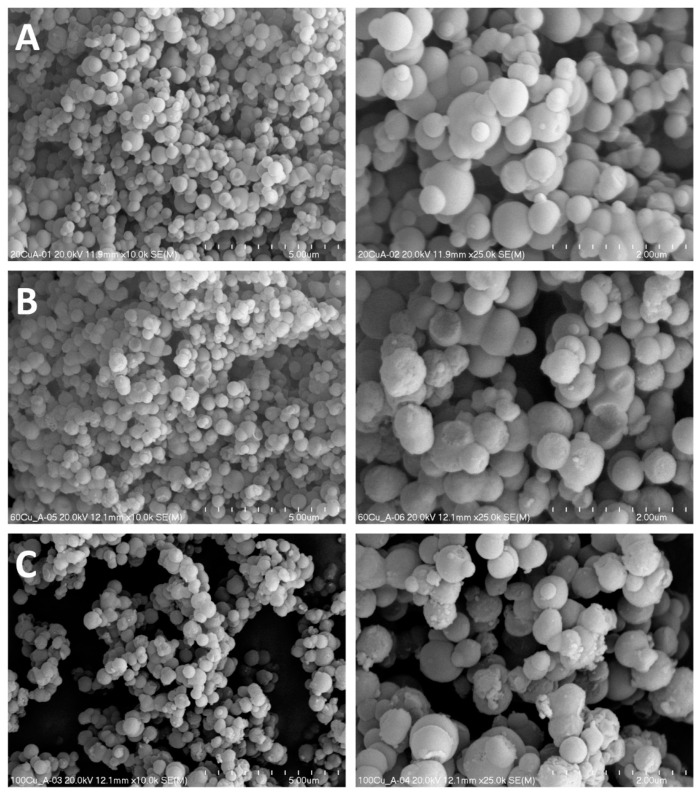
SEM images of the 20Cu-A (**A**), 60Cu-A (**B**) and 100Cu-A (**C**) samples at various magnifications.

Any significant changes in morphology were observed for the 20Cu sample modified with copper by TIE method (Figure 5A). An increase in copper loading in 60Cu did not result in any significant modification of size and distribution of silica-alumina spheres of MCM-41, but in this cases nanorods of CuO were identified in SEM images recorded for this samples (Figure 5B). The thickness of such nanorods is about 100–400 nm, while their length at least one order of magnitude larger. In the case of the 100Cu sample with the highest copper loading the presence of bulky CuO crystallites was found (Figure 5C). A similar effect was reported in our previous papers for the deposition of copper by the TIE method into pure silica spherical MCM-41 [12]. It seems possible that surfactants extracted from mesoporous silica are organised to form micellar structures in alcohol solution. Interaction of copper species with surfactant molecules in solution may result in the specific organisation of copper species, which under calcination conditions were transformed into CuO nanorods or bulky three-dimensional copper oxide crystallites. This interesting effect needs to be experimentally verified in the future. The SEM micrographs recorded for the samples modified with copper by TIE-NH_3_ method (Figure 6) show that treatment with ammonia solution resulted in a partial leaching of silica from the MCM-41 spheres and its deposition in the form of amorphous silica on the surface of spheres. This effect is more significant for the samples treated with more concentrated ammonia solution, 60Cu-A (Figure 6B) and 100Cu-A (Figure 6C).

In the PSD profiles of these samples, additional broad maximum in the range of 5–22 nm was observed (Figure 3B). It seems possible that such amorphous silica acts as kind of liners, which sticks MCM-41 spheres together. The spaces between such sticked spheres were possibly identified as large mesopores in the PSD profiles of 60Cu-A 100Cu-A. In the images of the samples obtained by TIE-NH_3_ method no nanorods and other CuO crystallites were identified (Figure 6). The results of SEM analysis are in full agreement with the XRD results (Figure 1) which show the presence of CuO crystallites only in the 60Cu and 100Cu samples (Figure 1A, insert).

**Figure 7:**
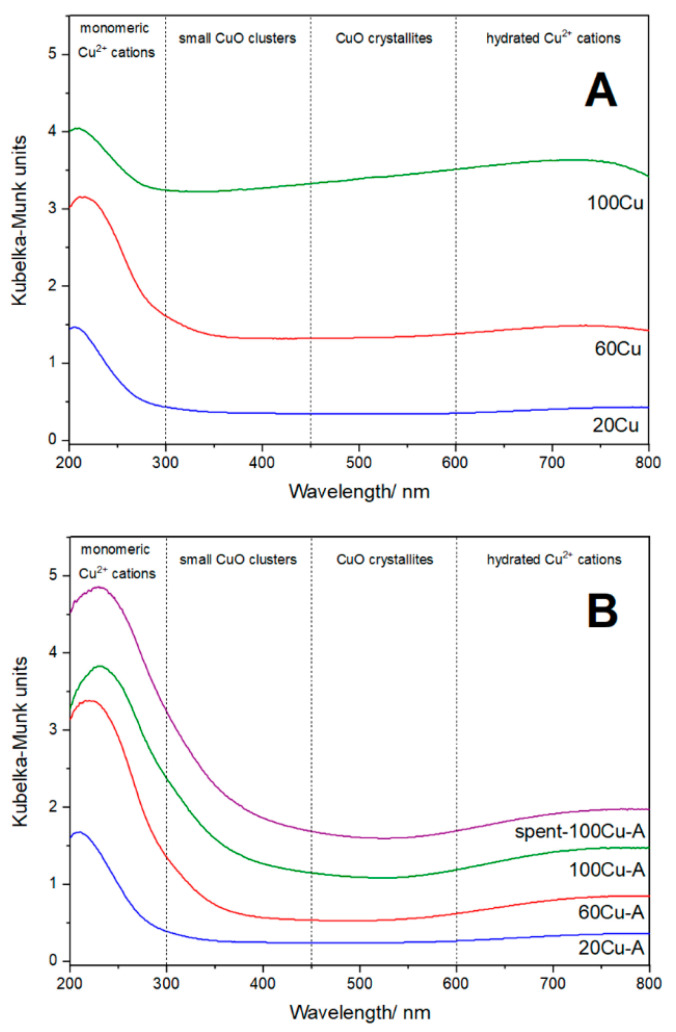
UV-vis-DR spectra of the Al-S-MCM-41 sample modified with copper by TIE (**A**) and TIE-NH_3_ (**B**) methods.

The form and aggregation of deposited copper species were analysed by UV-vis DR spectroscopy. The band centred at about 220–240 nm, dominating in the spectra of all copper modified samples (Figure 7A,B), is related to monomeric copper cations interacting with oxygen of silica (O^2−^→Cu^2+^). The presence of such monomeric copper ions is proved by the band 750–800 nm assigned to d-d transition of Cu^2+^ ions in pseudo-octahedral coordination (e.g., Cu(H_2_O)_6_^2+^) [15,16,17]. In a series of the samples obtained by the TIE-NH_3_ method, the intensity of the band characteristic of monomeric copper cations (220–240 nm) increased with an increase in copper loading (Figure 7B), indicating significant contribution of highly dispersed copper cations also in the samples with the higher content of this transition metal. In the case of the samples obtained by TIE method an increase in the intensity of this band is observed only for the 20Cu and 60Cu samples (Figure 7A). Meanwhile, the intensity of this band is significantly reduced in the spectrum of the sample with the highest copper loading, 100Cu, indicating a significant contribution of copper in the form of more aggregated species. The presence of such aggregated copper species in the 100Cu is proved by the increased absorbance level in the range of 300–450 nm characteristic of small oligomeric copper oxide species [17] and in the range of 450–600 nm indicating the presence of CuO crystallites [17]. Thus, the results of UV-vis DRS analysis of the samples are in line with the XRD studies, which showed the presence of CuO crystallites in the 100Cu sample (Figure 1A, insert). The reflections characteristic of the CuO crystallites were also found in diffractogram of 60Cu, however in this case the intensities of these diffraction peaks were significantly lower comparing to diffractogram of 100Cu.

The results of the XRD, SEM, and UV-vis DRS analysis clearly show that modified TIE method used for copper deposition on the MCM-41 spheres including treatment of the samples with ammonia solution resulted in its deposition mainly in highly dispersed form. The role of ammonia treatment of the copper modified samples is still not fully explained. However, it seems that ammonia molecules are bounded to copper cations by the formation of donor-acceptor bounds (accommodation of free electron pair of ammonia into unoccupied d-orbitals of copper cations). Such ammonia complexed surface copper species are possibly characterized by limited tendency to sintering. Another interesting effect is the formation of CuO nanorods in the samples obtained by TIE method (without ammonia treatment). Such CuO nanorods were not observed in the samples treated with ammonia solution directly after TIE procedures and prior calcination (TIE-NH_3_ series). Possibly precursors of such nanorods are formed in a solution by interaction of alkylammonium surfactants extracted from pores of MCM-41 and copper cations. Possibly, such surfactants interact with copper cations with the formation of ordered “micellar-like” type structures, which under calcination conditions are transformed into CuO nanorods. Treatment of the samples with ammonia solution, prior to the calcination of the samples (TIE-NH_3_ series), results in the formation of copper ammonia complex compounds in solution and therefore prevents the formation of CuO nanorods. This is the only hypothesis that must be verified by future studies.

**Figure 8:**
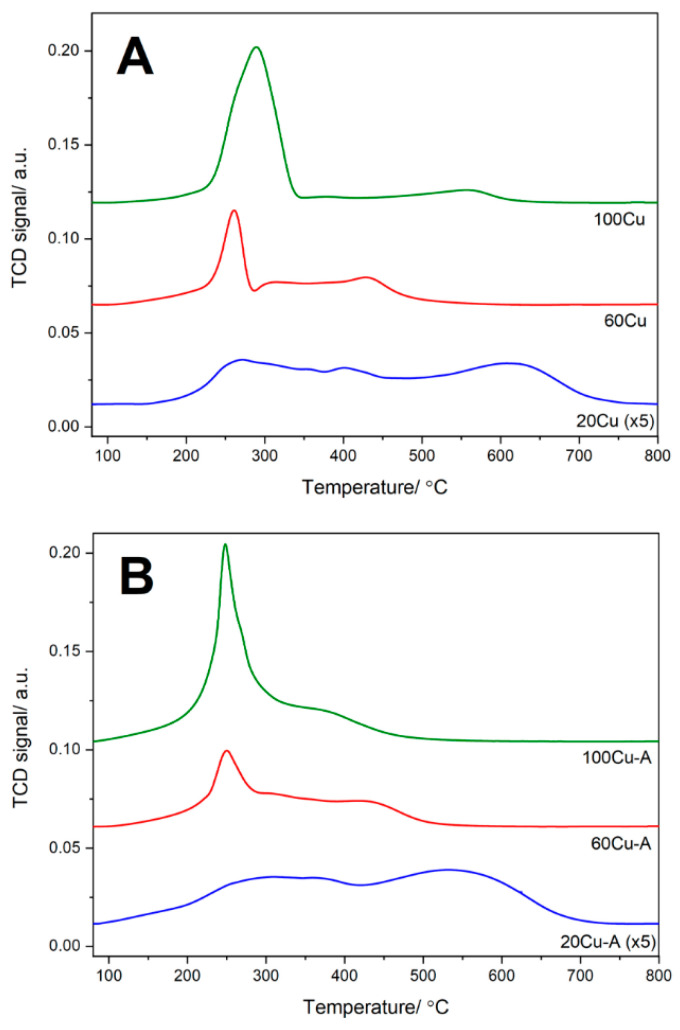
Results of H_2_-TPR studies of the Al-S-MCM-41 sample modified with copper by TIE (**A**) and TIE-NH_3_ (**B**) methods.

The reducibility of the copper modified samples was studied by the method of temperature-programmed reduction with using hydrogen as a reducing agent (H_2_-TPR). In the case of the copper containing samples the H_2_-TPR analysis may also give some information about form and aggregation of deposited copper species. The reduction of aggregated copper oxide species proceeds in one step, directly from of Cu^2+^ to Cu^0^, typically at temperatures below 300 °C [11,18]. On the other side the reduction of monomeric copper cations is separated for two steps. In the first step, below 300 °C, the Cu^2+^ cations are reduced to Cu^+^ ions. The reduction of monomeric Cu^+^ cations to Cu^0^ takes place at higher temperatures, typically above 300–350 °C [11] The analysis of the reduction profiles, presented in Figure 8, leads to the conclusion that the contribution of monomeric Cu^2+^ cations in the samples obtained by TIE method is about 97%, 69% and 21% in 20 Cu, 60Cu, and 100Cu, respectively. In the case of the samples of TIE-NH_3_ series, the contribution of such monomeric Cu^2+^ cations is significantly larger, at about 89% and 87% in 60Cu-A and 100Cu-A, respectively. In the case of 20Cu-A, the calculated value is above 100%, indicating that in this case part of copper exists as monomeric Cu^+^ cations. Possibly, under outgassing conditions part of copper cations in this sample was thermally reduced from Cu^2+^ to Cu^+^. Such effect has already been reported in scientific literatures [19]. Such a thermal reduction of Cu^2+^ to Cu^+^ under inert atmosphere (outgassing conditions) may suggest high lability in the changing of Cu^2+^→Cu^+^ oxidation state in this sample.

**Figure 9:**
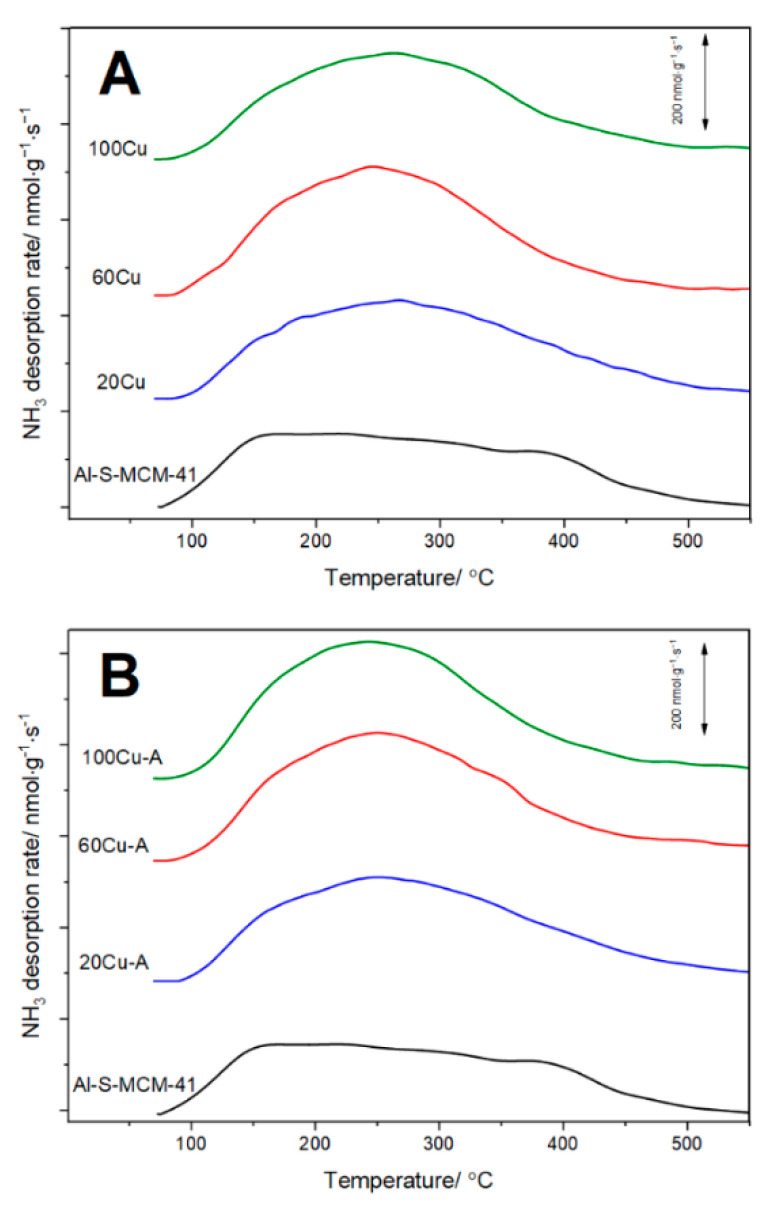
NH_3_-TPD profiles of the Al-S-MCM-41 sample modified with copper by TIE (**A**) and TIE-NH_3_ (**B**) methods.

The surface concentration of acid sites and their relative strength were analysed by the method of temperature-programmed desorption of ammonia (NH_3_-TPD). Ammonia desorption profiles are presented in Figure 9, while the surface concentration (C_a_) and surface density (D_a_) of acid sites in the samples are compared in Table 2. In the case of the Al-S-MCM-41 sample, the ammonia desorption profile is spread in a relatively broad temperature range and consists of at least three unresolved maxima, indicating significant heterogeneity of acid sites strength. In this sample acid sites are attributed to aluminum incorporated into silica wall of spherical MCM-41. The Al/C_a_ ratio in Al-S-MCM-41 is about 0.28, thus considering that one Al^3+^ ion could be assigned to one acid site, leads to the conclusion that only part of aluminum cations generates acid sites. Possibly part of aluminum is located inside silica walls and is not accessible for ammonia chemisorption and catalytic reaction. Deposition of copper by TIE method resulted in an increase of acid site concentration in the samples (Figure 9A, Table 2). Copper deposited on the support surface plays a role of Lewis acid sites and ammonia is bounded to this type of sites by donation of free electron pair into unoccupied d-orbitals of copper (NH_3_→Cu). Thus, the number of such copper sites depends on copper species loading and their surface accessibility. The concentration of surface acid sites (C_a_) gradually increased for 20 Cu and 60Cu but for 100Cu decreased below value determined for 60Cu (Figure 9A, Table 2). This result is in line with the results of XRD (Figure 1A, insert), UV-vis DRS (Figure 7A) and H_2_-TPD (Figure 8A) studies, which shown a significant contribution of aggregated copper oxide species in the 100Cu sample. Thus, in this case, part of the copper, located inside of such bulky aggregates, is not accessible for ammonia chemisorption and also for catalysis. In a series of the samples obtained by the TIE-NH_3_ method the surface concentration of acid sites (C_a_) increased with an increase in copper loading, indicating that the high dispersion of the deposited copper species (Figure 9B, Table 2). The comparison of the ammonia desorption profiles of Al-S-MCM-41 and its modifications shows that deposition of copper resulted in an increased ammonia desorption in the range of 150–400 °C. Thus, possibly in this range, ammonia bonded to copper cations desorbing from the samples.

**Figure 10:**
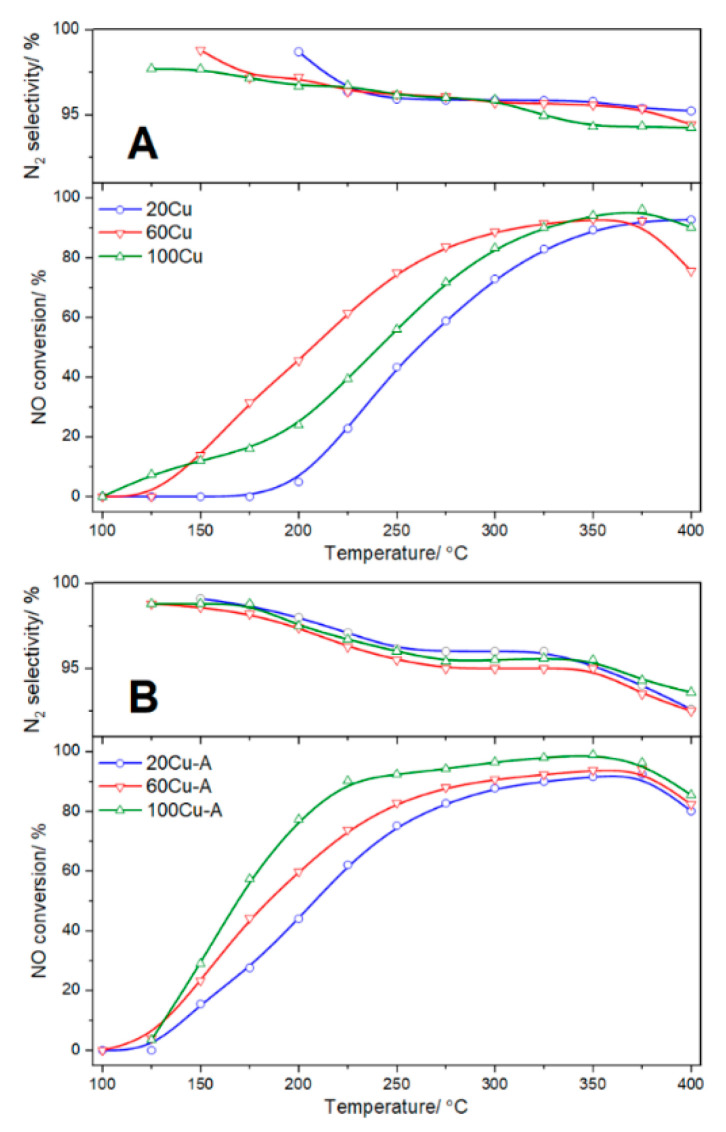
Temperature dependence of the NO conversion and N_2_ selectivity in NH_3_-SCR for the Al-S-MCM-41 sample modified with copper by TIE (**A**) and TIE-NH_3_ (**B**) methods.

**Figure 11:**
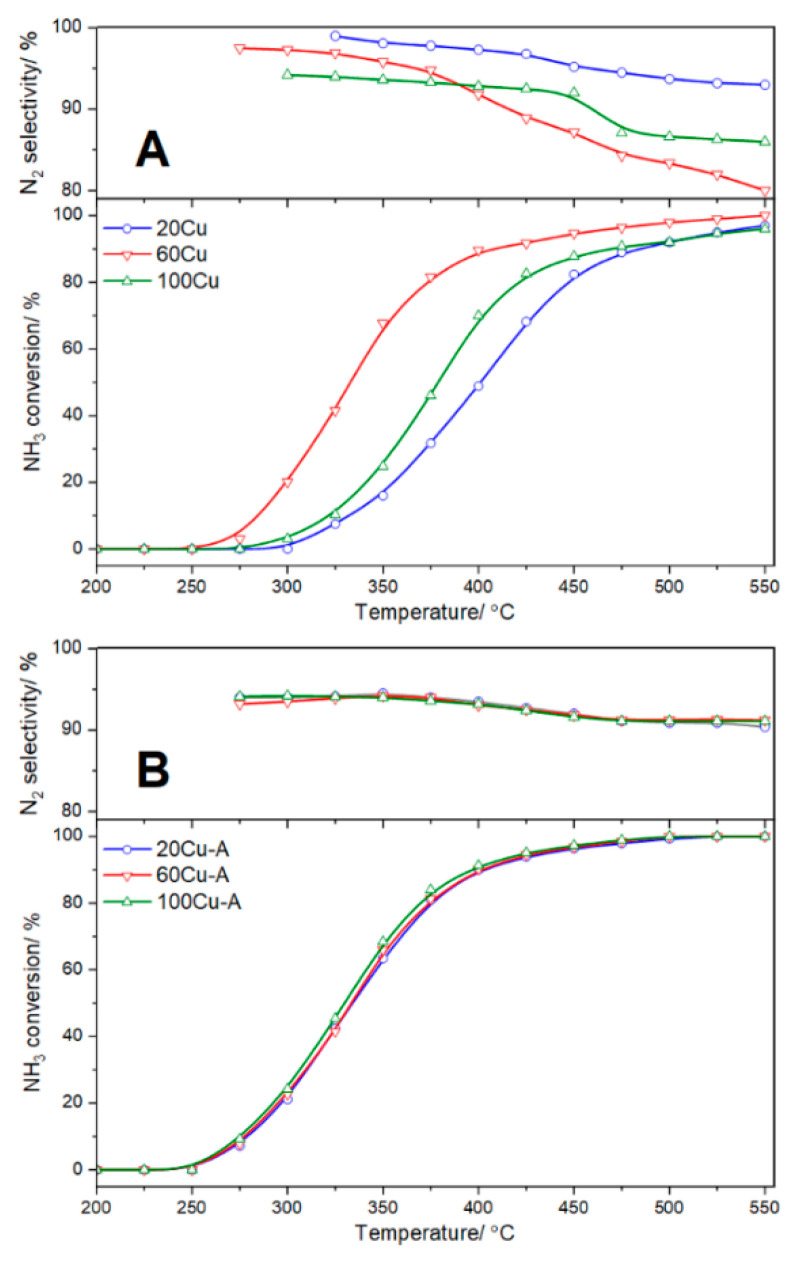
Temperature dependence of the ammonia conversion and N_2_ selectivity in process of ammonia oxidation for the Al-S-MCM-41 sample modified with copper by TIE (**A**) and TIE-NH_3_ (**B**) methods.

The copper modified samples were tested as catalysts of selective catalytic reduction of NO with ammonia (NH_3_-SCR). The results of these studies, presented in Figure 10, show relatively high activity of the studied catalysts in this process. In a series of the catalysts obtained by TIE method the NO conversion is observed from about 200 °C for 20Cu, but the temperature of the NO conversion initiation decreased with an increase of copper loading to 150 and 125 °C for 60Cu and 100Cu, respectively (Figure 10A). The NO conversion in the presence of the 60Cu and 100Cu catalysts reached a level of 90–93% at about 325–350 °C. The catalytic activity increased with an increase in copper loading for the 20Cu and 60Cu samples, but the catalyst with the highest copper loading, 100Cu, was less active than 60Cu (Figure 10A). This is possibly assigned to the lower surface accessibility of copper cations in 100Cu, due to the presence of the aggregated copper oxide species in this sample. The decrease in the NO conversion observed for the 60Cu and 100Cu catalysts above 350–375 °C is assigned to the side reaction of direct ammonia oxidation by oxygen present in the reaction mixture. In the case of 20 Cu, no decrease in the NO conversion was observed in the studied temperature range. The results of NH_3_-SCR catalytic tests are fully consistent with the results of ammonia oxidation over the catalysts of TIE series, presented in Figure 11A. As it can be seen efficiency of ammonia oxidation increased with an increase of copper loading for the 20 Cu and 60Cu catalysts but decreased for the samples with the highest copper loading, 100Cu, possibly due to limited accessibility of copper cations in bulky copper oxide species present in this sample. It should be noted the high selectivity to nitrogen, both in the NO reduction with ammonia as well ammonia oxidation. In the series of the catalysts obtained by TIE-NH_3_ method their activity increased with an increase of copper loading (Figure 10B). In the case of all catalysts of this series the NO conversion started from about 125 °C and increased with reaction temperature increase. In this series of the catalysts most copper was deposited in the form of monomeric copper cations and therefore accessibility of such copper sites was much better comparing to TIE series. In the case of the best catalyst of this series, 100Cu-A, the NO conversion above the level of 90% was obtained in the broad temperature range of 225–375 °C (Figure 10B). At temperature above 350 °C, the NO conversion decreased due to the side process of direct ammonia oxidation.

For the most active catalyst, 100Cu-A, stability under reaction condition was verified by three subsequent catalytic tests conducted in the temperature range from room temperature to 550 °C. Any significant differences in the NO conversion and N_2_ selectivity profiles were observed (results presented in Supplementary Materials), indicating stability of this catalyst under reaction conditions. Moreover, the catalyst sample after catalytic test was analysed with respect to form and aggregation of copper species (UV-vis-DRS, XRD), copper loading (ICP-OES) and textural properties (low-temperature N_2_ sorption). The UV-vis-DR spectra recorded for fresh and spent 100Cu-A catalyst are very similar (Figure 7A) indicating that the form of copper species was not changed under reaction conditions. It is proved by the lack of the reflection characteristic of CuO in the diffractogram of the sample after catalytic test (Figure 1A). The increase in BET surface area observed for the spent catalyst (Table 2) is in the accuracy range of BET method, which is estimated to be about 10%. Thus, the comparison of the characterization results determined for fresh and spent 100Cu-A catalysts clearly shows its high stability under reaction conditions.

The comparison of the NO conversion profiles (Figure 10B) and ammonia oxidation results (Figure 11B) leads to a very interesting observation. The copper loading strongly differentiated activity of the catalysts in the NH_3_-SCR process, while the profiles of ammonia oxidation are very similar and very slightly dependant on the copper content in the samples of this series. This interesting effect should be explained by the future studies. The selectivity to nitrogen in the NH_3_-SCR catalytic tests is very high and did not doped below 92% at temperatures lower than 400 °C (Figure 11B). Even more promising results were obtained in direct ammonia oxidation, where the selectivity to nitrogen was above the level of 90% up to temperature of 550 °C (Figure 11B). Thus, the studied catalysts obtained by TIE-NH_3_ method are not only effective catalysts for the NH_3_-SCR process, but also very promising catalysts for the selective ammonia oxidation to nitrogen.

**Figure 12:**
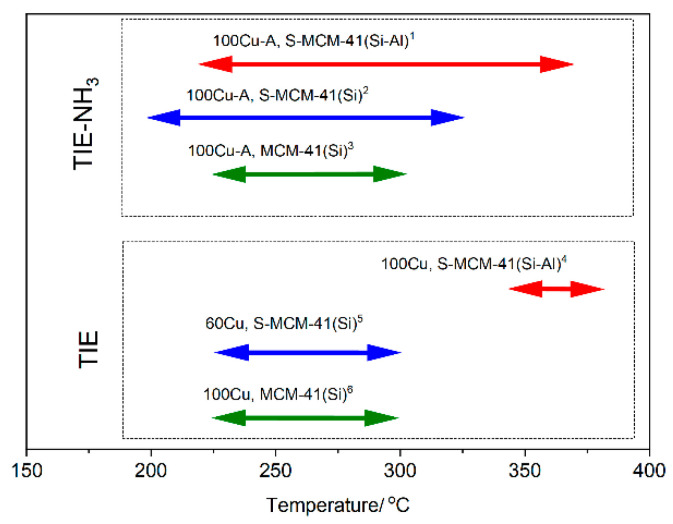
Comparison of the temperature ranges of the NO conversion over 90% for the most active catalysts obtained by TIE-NH_3_ and TIE methods: ^1^ silica-alumina spherical MCM-41 modified with copper by TIE-NH_3_ method, selectivity to N_2_ (S_N2_) in this range above 95%, this studies; ^2^ pure silica spherical MCM-41 modified with copper by TIE-NH_3_ method, S_N2_ > 98% [11]; ^3^ pure silica spherical MCM-41 modified with copper by TIE-NH_3_ method, S_N2_ > 97% [12]; ^4^ silica-alumina spherical MCM-41 modified with copper by TIE method, S_N2_ > 95%, this work; ^5^ pure silica spherical MCM-41 modified with copper by TIE method, S_N2_ > 98% [11]; ^6^ pure silica spherical MCM-41 modified with copper by TIE method, S_N2_ > 97% [12].

The temperature ranges of the NO conversion over 90% for the most active catalysts obtained by TIE-NH_3_ and TIE methods in current and previous studies [11,12] are compared in Figure 12. The catalytic tests for all the samples presented in this figure were done under the same conditions. First of all, a very significant difference in the temperature ranges of effective catalytic operation between the samples obtained by TIE and TIE-NH_3_ methods can be observed. Treatment of the samples with ammonia solution directly after TIE significantly extended these ranges for the pure silica and silica-alumina spherical MCM-41 based catalysts. This effect was not observed for the catalysts based on classical MCM-41 (Figure 12). Another important observation is assigned to the introduction of aluminum into spherical silica MCM-41, which slightly shifted the NO conversion to higher temperature but also significantly extended temperature window of effective operation in the NH_3_-SCR process. The shift in the low-temperature operation is possible assigned to competitive adsorption of ammonia molecules on Al-acid sites, which are inactive in their catalytic activation, and copper sites active in ammonia activation for the NH_3_-SCR reaction. In the case of pure silica supports ammonia can be chemisorbed only on the copper sites. The extended activity of the catalysts based on alumina doped mesoporous silica in the NH_3_-SCR process is related to the lower activity of these catalysts in ammonia oxidation [12]. Possibly part of ammonia molecules is chemisorbed on Al-acid sites, which in contrast to copper cations, stabilise ammonia against its oxidation. Of courses this is only hypothesis that should be proved by future studies. It should be noted that, independently of the MCM-41 morphology as well as the presence or absence of alumina, the deposition of copper by the TIE-NH_3_ method resulted in the catalysts being very selective in NO to N_2_ reduction.

## 3. Materials and Methods

### 3.1. Catalysts Preparation

#### 3.1.1. Synthesis of Al-S-MCM-41 Support

The procedure used for the synthesis of spherical silicon-aluminum MCM-41, denoted as Al-S-MCM-41, was reported by Szegedi et al. [20]. In the first step, hexadecyltrimethylammonium bromide (CTAB, Sigma-Aldrich, St. Louis, MO, USA), used as structure directing agent, was introduced into the mixture of distilled water, aqueous solution of ammonia (Avantor/POCH, Gliwice, Poland) and ethanol (Chempur, Karlsruhe, Germany), and then stirred at room temperature for 15 min. Then, sodium aluminate (NaAlO_2_, Sigma-Aldrich, St. Louis, MO, USA) and tetraethyl orthosilicate (TEOS, Sigma-Aldrich, St. Louis, MO, USA), used as aluminium and silicon sources, respectively, were added to the reaction mixture. The final composition of the reaction mixture to obtain Al-S-MCM-41 with the intendent molar Si/Al ratio of 20 contained the following molar ratio of the reactants: 1 TEOS:0.05 NaAlO_2_:0.3 CTAB:11 NH_3_:58 ethanol:144 H_2_O. The reaction mixture was intensively stirred at room temperature for 2 h and then the resultant slurry was filtered, washed with distilled water (to obtain pH = 7) and dried overnight at 60 °C. The obtained template-containing material is denoted as pre-Al-S-MCM-41. Calcination of pre-Al-S-MCM-41 at 550 °C for 6 h in air atmosphere resulted in surfactant removal.

#### 3.1.2. Deposition of Copper by Template-Ion Exchange Method (TIE)

Freshly synthesized pre-Al-MCM-41 material containing organic surfactants inside pores was modified with copper by template-ion exchange method (TIE). The extraction of the organic template with simultaneous deposition of metal was performed by stirring of non-calcined pre-Al-S-MCM-41 with methanol (VWR, Radnor, Pennsylvania, USA) solutions of copper chloride—CuCl_2_ (POCH, Gliwice, Poland). The concentration of CuCl_2_ solutions used for the TIE method was dependant on the assumed percentage extraction of template (Table 1). The pre-Al-S-MCM-41 sample, dispersed in copper-methanol (1 g/50 mL) solution, was stirred (500 rpm, magnetic stirrer) under reflux at 70 °C for 3 h. Then the samples were filtered, washed with methanol, dried at 60 °C overnight and finally calcined at 550 °C for 6 h in air atmosphere.

#### 3.1.3. Deposition of Copper by Modified Template-Ion Exchange Method (TIE-NH_3_)

The TIE-NH_3_ method is extended for treatment of the samples, directly after TIE procedure, with ammonia solution. Thus, directly after filtration and washing with methanol, the obtained sample was re-dispersed in an aqueous solution of ammonia (Avantor/POCH, Gliwice, Poland), 100 mL, and intensively agitated for 1 h. The concentrations of ammonia solution were individually chosen for each copper modified sample and were four times higher than concentration of copper in solutions used in the TIE procedure (Table 1). Then, the resulting solids were filtered, washed with distillate water, dried at 60 °C overnight and finally calcined at 550 °C for 6 h in air atmosphere.

The obtained samples are denoted as *x*Cu and *x*Cu-A, where *x* is the intended percentage of surfactants exchanged for copper, while A indicates the samples treated with ammonia solutions directly after TIE modification (TIE-NH_3_ method). The sample codes and concentrations of the reactant solutions used for their synthesis are presented in Table 1.

### 3.2. Catalysts Characterization

The X-ray diffraction patterns of the studied samples were recorded using a Bruker D2 Phaser (Bruker, Billerica, MA, USA) diffractometer. The measurements were performed in the low 2 Θ angle range of 1–7° and high 2 Θ angle range of 28–42° with a step of 0.02°. The counting time of 5 s per step and 1 s per step was used for the low-angle and high-angle measurements, respectively.

The chemical compositions of the samples (Si, Al, Cu) were determined by inductively coupled plasma optical emission spectrometry Thermo Scientific, Waltham, MA, USA). The solid samples were dissolved in a mixture containing 6 mL HNO_3_ (67–69%, Honeywell, Charlotte, NC, USA), 2 mL HCl (30%, Honeywell, Charlotte, NC, USA), and 2 mL HF (47–51%, Honeywell, Charlotte, NC, USA) at 190 °C using a microwave digestion system (Ethos Easy, Milestone, Sorisole, Italy).

Textural parameters of the samples were determined by N_2_-sorption at −196 °C using a 3Flex v.1.00 (Micromeritics, Norcross, GA, USA) automated gas adsorption system. Prior to the analysis, the samples were outgassed under vacuum at 350 °C for 24 h. The specific surface area (S_BET_) of the samples was determined using the BET model. The profiles of pore size distribution (PSD) were determined by the analysis of the adsorption branch of isotherm using the BJH model, while the pore volume was estimated by means of the total amount of adsorbed nitrogen at the relative p/p_0_ pressure of 0.98.

SEM images of the samples were recorded using Hitachi S-4700 (Hitachi Instruments Inc., San Jose, CA, USA) scanning electron microscope equipped with a Noran Vantage analyser.

The UV-vis DR spectroscopy was used for the analysis of the form and aggregation of copper species introduced into the spherical aluminum-silicon porous materials. The spectra were recorded using spectrometer Lambda 650S (PerkinElmer, Waltham, MA, USA) in the range of 200–800 nm with a resolution of 2 nm.

The method of temperature-programmed desorption of ammonia (NH_3_-TPD) was used for the evaluation of the surface acidity of the samples. The measurements were performed in a flow quartz microreactor system equipped with quadrupole mass spectrometer (QMS, PREVAC, Rogów, Poland) as detector. The flow rate and composition of gas mixture was adjusted and controlled by mass flow controllers (Brooks Instruments, 19440-0903 Hatfield, PA, USA). The sample of 50 mg was placed into microreactor and outgassed in a flow of pure helium at 550 °C for 30 min. Subsequently, the microreactor was cooled to 70 °C and the sample was saturated in a flow of gas mixture containing 1 vol.% NH_3_ diluted in helium for about 2.5 h. Then, the catalyst was purged in a helium flow until a constant base line level was attained. Ammonia desorption was carried out with a linear heating rate of 10 °C·min^−1^ in a flow of pure helium (20 mL·min^−1^). The calibration of the QMS detector with commercial mixture allowed recalculating the detector signal into the rate of ammonia desorption.

Reducibility of the samples was analysed by temperature-programmed reduction using H_2_ as reducing agent (H_2_-TPR). The measurements were carried out in a fixed-bed flow microreactor system equipped with thermal conductivity detector (TCD, Valco, Houston, TX, USA). The flow rate and composition of gas mixture was adjusted and controlled by mass flow controllers (Brooks Instruments, Hatfield, PA, USA). Prior to the H_2_-TPR runs, each sample (50 mg) was outgassed in a flow of pure argon at 550 °C for 20 min. After cooling down to 80 °C the H_2_-TPR runs were carried out in the range of 80–800 °C with the linear heating rate of 10 °C·min^−1^ in a flow of gas mixture containing 5.0 vol.% H_2_ diluted in argon (total flow rate of 10 mL·min^−1^).

### 3.3. Catalytic Studies

Mesoporous aluminum-silicon samples modified with copper were studied as catalysts of selective catalytic reduction of NO with ammonia (NH_3_-SCR) and selective catalytic oxidation of ammonia (NH_3_-SCO). Catalytic tests for both reactions were carried out in a flow fixed-bed quartz microreactor under atmospheric pressure. The flow rate and composition of gas mixture was adjusted and controlled by mass flow controllers (Brooks Instruments, 19440-0903 Hatfield, PA, USA). The reactant concentrations were continuously monitored using a quadrupole mass spectrometer (QMS, PREVAC, Rogów, Poland) connected directly to the reactor outlet. Prior to the catalytic tests, both in NH_3_-SCR and NH_3_-SCO reactions, the sample of 100 mg with the particle’s sizes in the range of 0.250–0.315 mm was placed in the quartz microreactor and outgassed in a flow of pure helium at 550 °C for 30 min. For the NH_3_-SCR runs the gas mixture containing 0.25 vol.% NO, 0.25 vol.% NH_3_ and 2.5 vol.% O_2_ diluted in pure helium (total flow rate of 40 mL·min^−1^) was used. The reaction was studied in the temperature range of 100–400 °C with intervals of 25 °C. In the case of NH_3_-SCO, the reaction mixture containing 0.5 vol.% NH_3_ and 2.5 vol.% O_2_ diluted in pure helium (total flow rate of 40 mL·min^−1^) was used and the reaction was studied in the range of 100–550 °C with intervals of 25 °C.

## 4. Conclusions

Spherical alumina-silica MCM-41 modified with copper by the template ion exchange method (TIE) and its modified version, including the treatment of the samples with ammonia solutions directly after copper deposition (TIE-NH_3_), was found to be an active catalyst for the selective reduction of NO with ammonia (NH_3_-SCR). In the case of the TIE method, part of the copper was deposited in the form of more aggregated copper species, such as CuO nanorods or bulky crystallites, which were less active in the NH_3_-SCR reaction. On the other hand, the deposition of copper into spherical alumina-silica MCM-41 by TIE-NH_3_ method very effectively limited formation of aggregated copper oxide species and therefore resulted in much more efficient catalysts for the NH_3_-SCR process. The comparison of the catalytic performance of the copper containing catalysts based pure silica and alumina-silica spherical MCM-41 shows that the presence of alumina slightly shifted the NO conversion profile into higher temperatures but also extended temperature window of the effective catalytic operation into higher temperatures. The selectivity of the studied catalysts towards nitrogen, independent of the method used for copper deposition (TIE or TIE-NH_3_), is above 92% at temperatures up to 400 °C. The reduction of NO with ammonia is limited at higher temperatures by direct ammonia oxidation by oxygen present in the reaction mixture. The additional studies of ammonia oxidation showed relatively high activity of the studied catalysts in this reaction which, however, were lower for the catalysts based on alumina-silica MCM-41than for the analogous catalysts based on pure silica MCM-41. Thus, alumina incorporation into silica spheres decreased efficiency of the side process of ammonia oxidation and therefore improved operation of these catalysts in the NH_3_-SCR process at higher temperatures. The studies of the ammonia oxidation process in the presence of the catalysts of TIE-NH_3_ series showed a very high selectivity to nitrogen (above 90% up to temperature of 550 °C). Thus, spherical alumina-silica MCM-41 modified with copper by TIE-NH_3_ method seems to be a very promising catalyst not only of the NH_3_-SCR process, but also selective ammonia oxidation to nitrogen.

## Figures and Tables

**Table 1:** The sample codes and amounts of reactants used for their synthesis.

Sample Code	Weight of Al-MCM-41 */g	Solution Volume/cm^3^	CuCl_2_ Concentration/mmol	NH_3_ Concentration/mmol
20Cu	2	100	0.35	-
60Cu	2	100	1.04	-
100Cu	2	100	1.74	-
20Cu-A	2	100	0.35	1.39
60Cu-A	2	100	1.04	4.16
100Cu-A	2	100	1.74	6.96

* non-calcined Al containing MCM-41.

**Table 2:** Textural parameters, chemical composition, and surface acidity of the copper modified Al-S-MCM-41 samples.

Sample Code	S_BET_/m^2^·g^−1^	Pore Volume/cm^3^·g^−1^	Pore Diameter/nm	Si/Al/mol·mol^−1^	Cu/wt%	* C_a_/μmol·g	** D_a_/μmol·m^−2^
Al-S-MCM-41	1194	0.755	3.1	27	-	247	0.207
20Cu	1132	0.700	3.1	27	1.9	313	0.277
60Cu	1097	0.553	3.0	29	5.0	345	0.314
100Cu	1003	0.587	3.1	37	6.0	315	0.314
20Cu-A	1139	0.705	3.1	27	1.9	346	0.304
60Cu-A	662	0.648	3.0	25	5.4	390	0.589
100Cu-A	589	0.605	3.0	26	7.9	396	0.672
spent 100Cu-A	635	0.532	3.0	26	7.9	-	-

* C_a_—surface concentration of acid sites related to 1 g of the sample; ** D_a_—surface density of acid sites—concentration of acid sites on 1 m^2^ of the sample.

## Data Availability

Data available on request.

## Supplementary Materials

The following are available online. Figure S1: Temperature dependence of the NO conversion and N_2_ selectivity in NH_3_-SCR for the 100Cu-A sample performed in the 3 cycles.
