# Supplementary material for: Spherical Al-MCM-41 Doped with Copper by Modified TIE Method as Effective Catalyst for Low-Temperature NH3-SCR
Source: Molecules. 2021 Mar 23;26(6):1807. doi: 10.3390/molecules26061807 (PMC8005196; doi:10.3390/molecules26061807)
Supplement: Supplementary file 1 [file molecules-26-01807-s001.pdf]

Supplementary Materials

Spherical Al-MCM-41 doped with copper by modified TIE method as effective catalyst for low-temperature NH_3-SCR

Aleksandra Jankowska[1], Andrzej Kowalczyk[1], Małgorzata Rutkowska[1],

Marek Michalik[2], Lucjan Chmielarz[1*]

[1]Jagiellonian University in Kraków, Faculty of Chemistry, Gronostajowa 2, 30-387 Kraków, Poland

[2]Jagiellonian University in Kraków, Institute of Geological Sciences, Gronostajowa 3a, 30-387 Kraków, Poland

[*]Corresponding author. Tel.: +48 126862417, fax: +48 12 686 2750.

E-mail address: chmielar@chemia.uj.edu.pl (L. Chmielarz)

For the 100Cu-A sample, which was found to be the most NH_3-SCR catalyst in, additonal verification of its catalytic stability was done by three subsequent catalytuc cycles. The catalytic runs were carried out in a flow fixed-bed quartz microreactor under atmospheric pressure uder thes same conditions as in standard catalytic tests. The flow rate and composition of gas mixture was adjusted and controlled by mass flow controllers (Brooks Instruments, 19440-0903 Hatfield, PA, USA). The reactant concentrations were continuously monitored using a quadrupole mass spectrometer (QMS, PREVAC, Rogów, Poland) connected directly to the reactor outlet. Prior to the catalytic tests, the sample of 100 mg with the particle's sizes in the range of 0.250-0.315 mm was placed in the quartz microreactor and outgassed in a flow of pure helium at 550ºC for 30 min. The gas mixture containing 0.25 vol.% NO, 0.25 vol.% NH_3 and 2.5 vol.% O_2 diluted in pure helium (total flow rate of 40 $mL{\cdot}min^{-1}$) was used. The reaction was studied in the temperature range from room temperature to 550ºC with intervals of 25ºC (only te region from 100 to 400°C is presnted).

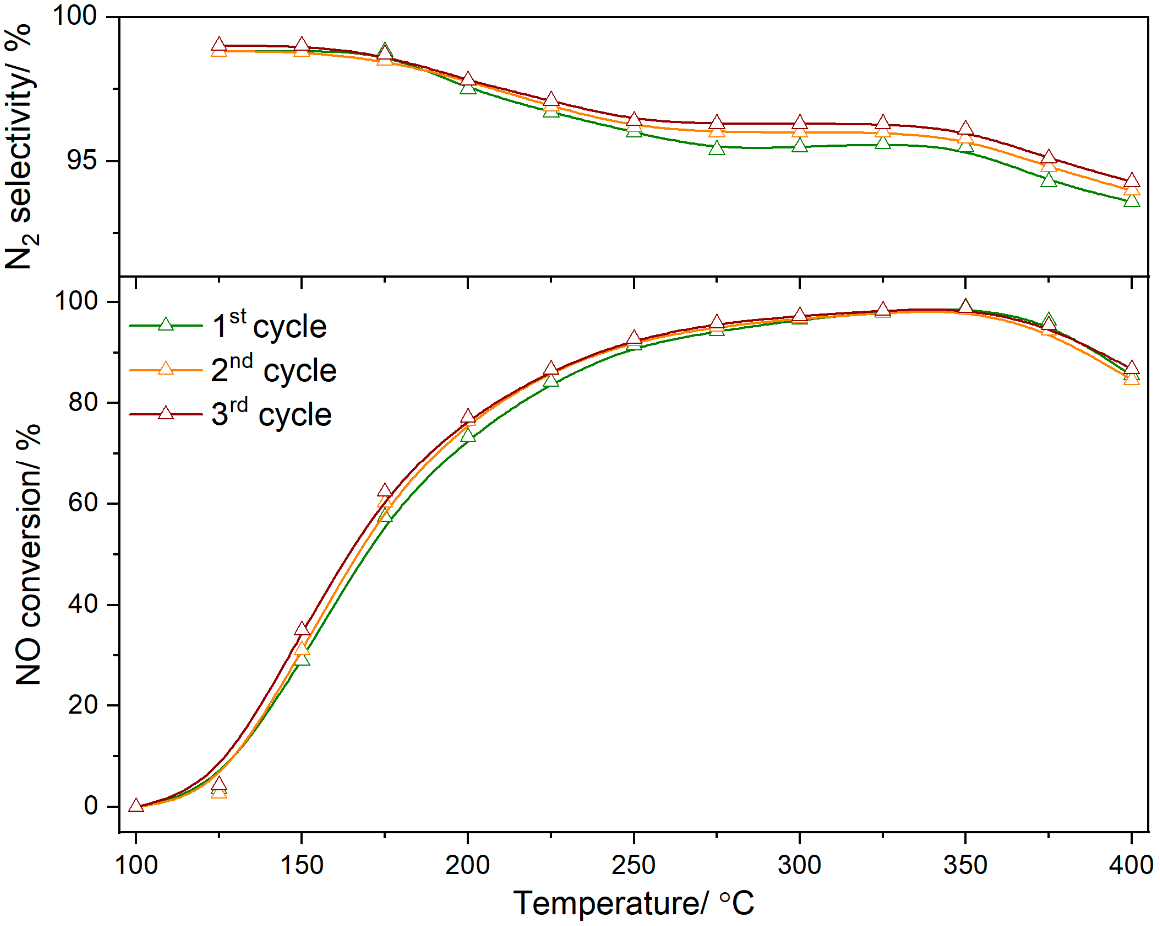

Figure S1. Temperature dependence of the NO conversion and N_2 selectivity in NH_3-SCR for the 100Cu-A sample performed in the 3 cycles.
